# Hidden Hazards Revealed: Mycotoxins and Their Masked Forms in Poultry

**DOI:** 10.3390/toxins16030137

**Published:** 2024-03-06

**Authors:** Hamada Okasha, Bochen Song, Zhigang Song

**Affiliations:** 1 Key Laboratory of Efficient Utilization of Non-Grain Feed Resources, College of Animal Science and Technology, Shandong Agricultural University, Taian 271018, China; hamada.okasha@fagr.bu.edu.eg (H.O.); bochensong@sdau.edu.cn (B.S.); 2 Animal Production Department, Faculty of Agriculture, Benha University, Moshtohor 13736, Egypt

**Keywords:** mycotoxins, masked, chicken productivity, immunity, antioxidants, blood, internal organs

## Abstract

The presence of mycotoxins and their masked forms in chicken feed poses a significant threat to both productivity and health. This review examines the multifaceted impacts of mycotoxins on various aspects of chicken well-being, encompassing feed efficiency, growth, immunity, antioxidants, blood biochemistry, and internal organs. Mycotoxins, toxic substances produced by fungi, can exert detrimental effects even at low levels of contamination. The hidden or masked forms of mycotoxins further complicate the situation, as they are not easily detected by conventional methods but can be converted into their toxic forms during digestion. Consequently, chickens are exposed to mycotoxin-related risks despite apparently low mycotoxin levels. The consequences of mycotoxin exposure in chickens include reduced feed efficiency, compromised growth rates, impaired immune function, altered antioxidant levels, disturbances in blood biochemical parameters, and adverse effects on internal organs. To mitigate these impacts, effective management strategies are essential, such as routine monitoring of feed ingredients and finished feeds, adherence to proper storage practices, and the implementation of feed detoxification methods and mycotoxin binders. Raising awareness of these hidden hazards is crucial for safeguarding chicken productivity and health.

## 1. Introduction

Mycotoxins have been recognized as a longstanding threat to animal health, produced by fungi *Aspergillus*, *Fusarium*, and *Penicillium*. While extensive research has been conducted, our understanding of the impact of these complex substances and their modified forms on poultry, which are highly vulnerable, remains limited. Effects on poultry include reduced feed intake, growth performance, immunity, antioxidants status, blood parameters, and organ damage. Mycotoxins and their masked forms have been associated with increased mortality, carcinogenicity, teratogenicity, and organ damage [1]. The significance of mycotoxins on public health is still a topic of ongoing discussion, beyond their negative influences on poultry productivity and well-being.

Mycotoxins exhibit structural variations due to their diverse origins, resulting in significant differences in their physical, chemical, and biological properties [2]. Aflatoxin B1 (AFB1), deoxynivalenol (DON), fumonisin Bs (FBs) and zearalenone (ZEN) are commonly encountered mycotoxins that often co-occur in grains and animal feeds. These mycotoxins, including AFB1, DON, and ZEN, are widespread in agricultural products and animal feed [3]. Rychlik et al. [4] AFB1 is considered the most potent and harmful aflatoxin, produced as a secondary metabolite by *Aspergillus flavus* and *Aspergillus parasiticus* fungi. This mycotoxin is recognized for its detrimental effects on both humans and animals [5,6,7]. When AFBI is encountered, the liver is the primary organ impacted [8,9]. This poses considerable challenges to the body’s immune response and gut health. ZEN is a mycotoxin produced by *Fusarium* sp. that has estrogenic properties and can lead to reproductive disorders [10]. Furthermore, mycotoxins can elicit the formation of reactive oxygen species (ROS), leading to oxidative stress and the oxidation of DNA, proteins, and lipids [11]. Additionally, the simultaneous presence of mycotoxins leads to their combination and the resulting synergistic toxic impact, significantly compromising the well-being and productivity of animals. Consequently, this situation leads to substantial economic losses in the field of animal husbandry [4,12].

The term of “masked mycotoxin” was introduced to describe cases of mycotoxicosis that did not correlate with the detected presence of certain mycotoxins. Masked mycotoxins undergo changes in structure, polarity, solubility, and molecular mass. Conventional techniques used to detect mycotoxins may not effectively identify masked mycotoxins, making it challenging to accurately estimate the total amount of mycotoxins in contaminated feed. Although limited toxicological data is available, there is a potential risk to human and animal health as masked mycotoxins can convert into their free form and enhance bioavailability [13,14,15]. Animal feeds often contain both masked and free mycotoxins [16,17]. Given the significant impact of the poultry industry on human health and well-being through the supply of animal protein, this concern is justified.

Therefore, it is crucial to have a comprehensive understanding of the composition and analysis of both free and masked mycotoxins. The focus of this review is to provide insights into the impact of mycotoxins, including their hidden forms, on chickens. Specifically, it examines how these substances affect nutritional efficiency, production performance, immune status, antioxidant status, and biochemical properties of chicken blood. Additionally, it explores the effects of mycotoxins on the internal organs of these birds. Therefore, it is beneficial to concentrate on studying mycotoxins and their various metabolites, which have not been thoroughly investigated, in terms of their productive and metabolic capabilities, especially in poultry. This will be advantageous for future identification of such attributes in both field and laboratory settings.

## 2. Masked Mycotoxins

Studying “masked and/or hidden mycotoxins,” which are plant metabolites of mycotoxins, or as defined by Rychlik et al. [18]. as “biologically modified,” mycotoxins, presents unique challenges. This is because the chemical modifications introduced by the plant’s metabolism can potentially impact both the toxicity of the mycotoxin (which may increase or decrease compared to the original toxin molecule) and its detectability through analytical methods. Masked toxins, in the case of the latter, are either attached to carbohydrates or proteins, making them unable to be extracted using current protocols designed for toxin extraction. Alternatively, these toxins may not be identifiable through established chromatography routines, which is why they are referred to as “masked or hidden” mycotoxins [19]. In addition, due to their structural similarities, certain masked compounds, which may have varying levels of toxicity, can be simultaneously detected with the toxin through methods such as immunoassays [13]. The lack of data remains a prevailing issue due to the challenges posed by analysis and the subsequent absence of established methodologies for routine testing [20]. ZEN-14-sulfate and DON-3-glucoside are frequently detected in feed as the most prevalent masked mycotoxins. Ongoing research is focused on understanding their toxicological characteristics, particularly the transformation of DON-3-glucoside into DON and ZEN-14 sulfate into ZEN by the microbiota residing in the intestinal tract [21].

Certain mycotoxins, including deoxynivalenol, T-2, and HT-2, have the ability to bind with proteins or carbohydrates, leading to alterations in their structures depending on the conditions. These particular forms of mycotoxins, where they are bound or conjugated with other components, are referred to as “masked mycotoxins” [18]. Despite the introduction of various techniques for detecting and measuring mycotoxins in different food matrices, such as immunochemical and chromatographic techniques, conventional analytical methods often struggle to detect or identify the derivatives or masked forms of mycotoxins [13,18,22,23]. The issue of feeds being contaminated by hidden mycotoxins has caused significant worries. These mycotoxins have a high level of bioavailability in the digestive system, particularly when they transform back into their original form [24]. Masked mycotoxins are modified versions of mycotoxins that cannot be identified using traditional analytical techniques because their structures have been altered within the plant [25]. While conventional methods like ELISA can detect masked forms, this is less likely with HPLC-based methods. It has been proposed that the analysis of mycotoxin levels in samples containing these compounds may result in an underestimation. The reason for the undetectability of masked mycotoxins during analysis lies in the changes in their physicochemical properties [13]. Recognizing the toxicological significance of masked mycotoxins in food products implies the need for the development of generic toxicity estimates that can be utilized by regulators and food producers to safeguard consumer health [26]. There are no existing legal regulations regarding safe levels of chemically differentiated mycotoxins in food and feed. Ongoing risk assessment studies are being conducted to address this issue [26]. Additionally, the toxicity of other fungal secondary metabolites, such as aurofusarin (AUR) and coulmorin (CUL), commonly found in cereals, remains unclear and is still under extensive research. Furthermore, there is a lack of information regarding the fate of masked, modified, and emerging mycotoxins and other secondary fungal metabolites in corn products and by-products, which are crucial raw materials in the food industry as well as for the production of animal feed [27].

These modified or masked mycotoxins coexist alongside their original forms [18]. Due to their intricate and fluctuating chemical composition, as well as their widespread existence, both humans and animals have the potential to come into contact with one or multiple mycotoxins. These mycotoxins can either be in their original form or undergo modifications when consumed through a contaminated diet. The occurrence of masked mycotoxins is more frequently observed in food as compared to feed. While there is some information available on the presence of ZEN and its modified forms resulting from phase I and phase II biotransformation, there is limited quantitative data on other modified forms such as acetyl DON derivatives, hydrolyzed FBs, and phase I metabolites T2 and NIV3G. Furthermore, the available data on masked mycotoxins is still insufficient and inconsistently reported, despite the growing recognition of their contribution to mycotoxin toxicity. It is crucial to take into account the masked mycotoxins in order to assess the risk of mycotoxins in poultry in the future [17,28]. Encouragingly, there have been recent advancements in analytical methods, indicating potential improvements in the simultaneous detection of multiple mycotoxins, both in their original and masked forms. However, the use of analytical methods still poses limitations in terms of cost and the absence of standardized protocols, hindering comprehensive data collection [29].

## 3. Major Types of Mycotoxins and Their Masked Forms in Chickens

### 3.1. Aflatoxin

It is important to consider the specific circumstances surrounding mycotoxin exposure in order to understand the potential effects on animals. The type of mycotoxin, the level and duration of exposure, as well as the age and species of the animal (Table 1), all play a role in determining the impact on their health [30]. Aflatoxins have been linked to various negative consequences in birds, such as impaired performance, weakened immune system, damage to organs, and decreased production of eggs [31,32]. The presence of fumonisin B1 (FB1) has been associated with the harmful effects on the chicken’s body’s ability to produce sphingosine (So) and sphinganine (Sa), which are important components of sphingolipids. This disruption occurs because FBs have a similar structure to these sphingolipids, leading to toxicity [28,33]. It should be acknowledged that aflatoxin, however, leads to an elevation in endogenous nitrogen loss, which could potentially be attributed to the shedding of the mucosal layer. This increase in mucin and proenzymes provides sufficient materials for the rapid turnover of proteins in the cells of the intestinal epithelium, particularly during aflatoxicosis. Additionally, it has been documented that FBs can induce nephrotoxicity, diarrhea, decreased body weight gain, and organ impairment in chickens [28,34,35]. AFB1, known as aflatoxin B1, possesses the highest level of toxicity and exhibits the most potent pathogenic impact among all the variants [36]. Poultry exhibit a remarkable sensitivity towards AFB1, as studies have revealed. The effects of AFB1 on poultry are contingent upon various factors such as the type, age, sex, and other relevant aspects of the poultry population [37,38]. The poisoned poultry exhibit several characteristic symptoms such as depression, reduced appetite, weight loss, and an unsteady gait. Among the different organs, the liver undergoes the most significant pathological changes. These changes are evident through the liver’s enlargement, rounded and blunt edges, brittle and fragile texture, pale yellow color, occasional increase in liver weight relative to body weight, and the presence of gray-white spots on its surface. Histopathological examination of the liver reveals extensive denaturation and necrosis of liver cells, disordered liver cords, hyperplasia of bile ducts, moderate hyperplasia of liver tissue, and the occurrence of liver fibrosis in chickens with a prolonged duration of the disease [28]. Nutrient availability can be influenced by various factors, and feed intake is certainly one of them. Mycotoxins in the livestock industry lead to decreased consumption of feed and inefficient utilization (Table 1), resulting in significant financial setbacks [39]. Rashidi et al. have documented the impact of different mycotoxins on feed intake. The inclusion of AFB1-contaminated diet resulted in an increased feed conversion ratio (FCR) for broiler chickens during both the grower (12–24 days) and finisher periods (25–42 days) [40]. The poultry industry suffers significant losses due to the negative impact of mycotoxins on the growth performance and health of poultry. This results in poor growth rates and various health problems (Table 1), leading to substantial financial setbacks for the industry. The findings [41] demonstrated that broilers fed diets containing excessive levels of AFB1, along with low levels of DON and ZEN, experienced a significant decline in their performance. Broiler chickens that were fed AFs at levels ranging from 50 to 200 µg/kg experienced a reduction in body weight and a decrease in body weight gain, as reported [10,42]. Birds that were given diets containing either 34 µg/kg or 500 µg/kg of AFB1 experienced a decrease in their body weight gain, in contrast to the birds that were fed diets free from AFs [43,44]. The growth performance of chickens can be influenced by various factors, including the varying concentrations of mycotoxins and their metabolite binders, the origin of mycotoxins, and the specific chicken species. These factors may have distinct impacts on the growth of chickens, highlighting the importance of considering these differences when assessing their growth performance.

The immunosuppressive effects of certain mycotoxins have been linked to decreased immune responses. Ochieng et al. [30] observed that mycotoxins, when administered in immunotoxic doses, have shown to have a lesser impact on bird performance compared to the doses required to induce a decrease in performance. The impact of AFB1 (at doses of 50 or 200 µg/kg feed) on the immune system of broiler chickens was examined in a feeding trial. Immunosuppression is a practical concern when it comes to higher levels of AFB1, as it has been observed that poultry feeds can contain levels of up to 1067 µg/kg [70]. The young layers that were fed 200 µg AFB1/kg feed and exposed to fowl adenovirus 4 experienced the highest mortality rate [71]. In a different research, it was found that broiler chickens that were given AFB1 (750 or 1500 µg/kg feed) and exposed to the *Clostridium perfringens* pathogen had the highest mortality rate [72]. Mycotoxins have been identified as having a detrimental effect on the functioning of antioxidant enzymes [73]. Chen et al. [62] reported that the spleen of chickens exhibited the production of oxidative stress following the ingestion of AFB1, as evidenced by the reduction in levels of antioxidant enzymes including GSH-Px, GR, CAT, as well as the levels of malondialdehyde (MDA) and GSH. In their study, Shahid et al. [63] discovered that the presence of 1 mg AFB1/kg diet in one-day-old chick diets led to an increase in MDA levels. Additionally, they observed a decrease in the activities of T-SOD, GSH-Px, CAT, GR, and GSTs in both liver and serum samples. These reduced enzyme activities could potentially contribute to the production of hydroxyl radicals, which are known to play a significant role in the process of lipid peroxidation [73]. The broilers that were fed a diet containing 1 ppm aflatoxin B1 exhibited an increase in the activity of SOD, while the activity of CAT decreased in comparison to the control group. Additionally, the levels of MDA in the serum of the broilers fed aflatoxin were found to be higher have been reported [61].

Oskoueian et al. [74] also reported similar results when they applied AFB1 mycotoxin in vitro to the hepatocytes of five-week-old roosters. The activity of antioxidant enzymes was adversely affected, while the levels of MDA (malondialdehyde) increased simultaneously. The findings [41] indicated that diets contaminated with mycotoxins resulted in notable increases in the levels of serum malondialdehyde (MDA) and 8-hydroxy-20-deoxyguanosine (8 OHdG). Additionally, the mRNA expressions of TLR4 and 4EBP1, which are linked to oxidative stress, were significantly affected. Stresses linked to mycotoxin exposure are thought to be mostly caused by disruption of redox equilibrium, signaling, and dysregulation of the antioxidant defense. Impaired protein synthesis has been found to result in alterations in blood parameters. These changes are characterized by a decrease in the levels of total protein, globulin, and albumin in the blood, and have been associated with mycotoxicosis. In a study involving broiler chickens, it was observed that feeding them with 200 µg AFB1/kg feed led to a reduction in plasma protein levels. Similarly, broiler chickens fed high levels of AFs (ranging from 2000 to 5000 µg/kg feed) exhibited decreased levels of serum total protein, albumin, and globulin [54]. Numerous studies conducted in laboratory settings and outside of living organisms have provided evidence that the consumption of feed contaminated with mycotoxins can result in organ damage in chickens (Table 1). This damage can be observed through changes in the weights of these organs, either an increase or decrease depending on the specific circumstances. The broilers that were fed diets contaminated with 500 µg AFB1/kg exhibited an elevation in the weights of their liver, spleen, and kidneys, as per the reported [44]. In addition, broilers that were given AFs at low (20 µg/kg feed), moderate (200 µg/kg feed) and, high (2000 µg/kg feed) levels exhibited an increase in the weights of their liver, heart, and kidney [75,76]. AFs predominantly accumulate in the liver after absorption for detoxification, making it the primary organ targeted by these substances. The liver is recognized as the main site where AFs accumulate and undergo detoxification processes, changes in liver weight are specifically linked to the level of AF in the diet [75].

**Figure 1:**
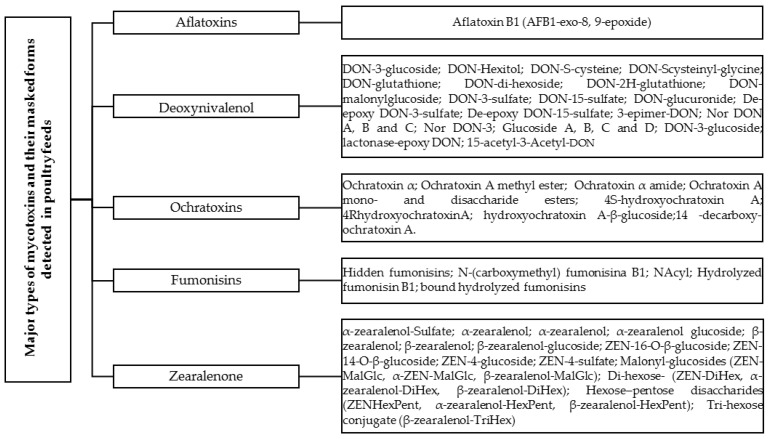
Diversity of Mycotoxins and Their Masked Forms in Poultry.

AFB1 and its masked form, AFB1-exo-8, 9-epoxide (Figure 1), are frequently encountered and exhibit potent carcinogenic, genotoxic, hepatotoxic, immunotoxic, and other detrimental effects in various animal species, including poultry [77]. AFB1, through its active intermediate product AFB1-exo-8, 9-epoxide, can bind with DNA, leading to the formation of the predominant trans-8, 9-dihydro-8-(N7-guanyl)-9-hydroxy-AFB1 (AFB1-N7-Gua) adduct. This adduct is responsible for causing DNA lesions [78]. AFB1-8, 9-exo-epoxide exerts a potent biological activity that can impact every phase of the cell cycle [79]. Studies have shown that AFB1 induces the arrest of chick jejunum cells at the G2/M phase [80], renal cells at the G0/G1 phase, and increases the percentage of chick thymocytes in the G2/M phase [81].

In addition, the intestine acts as a site for metabolic activation of aflatoxins and generates their masked forms, specifically AFB1-exo-8, 9-epoxide (AFBO). AFBO primarily targets rapidly dividing intestinal enterocytes characterized by high protein turnover. AFBO has the ability to inhibit protein synthesis by interacting with RNA and can also form DNA adducts, leading to DNA breakage. In addition, AFBO can induce epigenetic effects such as DNA methylation, histone modifications, the maturation of miRNAs, and the generation of single nucleotide polymorphisms on a daily basis [82]. Consequently, AFBO’s impact on DNA, RNA, and protein synthesis in the gastrointestinal tract (GIT) collectively influences enterocyte integrity, endogenous nutrient loss, nutrient digestion and absorption, and other intestinal functions [83]. AFB1, along with its masked form, can influence a range of genes, proteins, and enzymes involved in cell cycle regulation [84]. This includes mutations in the p53 gene and methylation of the p16 gene in tissue, which can impact the activities of CDK4, CDK6, and Rb [82]. Additionally, it can lead to a decrease in the number of PCNA-positive cells [84]. The contamination of AFB1 and its metabolites in the diet resulted in reduced chicken weight at 14 and 21 days, indicating a negative impact on growth performance. To assess the development status and extent of pathological changes in the spleen, the relative weight of the spleen was utilized. At 14 and 21 days of age, the AFB1 group exhibited a significantly lower relative spleen weight compared to the control group [78]. When AFB1 is present in poultry diets, it triggers the production of CYPP450 isoenzymes, resulting in the conversion of AFB1 to the highly toxic masked form known as AFB1-8, 9-epoxide. This transformation leads to oxidative damage, organ failure, decreased productivity, impaired reproductive performance, heightened susceptibility to diseases, and the accumulation of AFB1 in eggs and meat. These effects pose potential risks to the health of consumers. Furthermore, AFB1 also interferes with the accumulation of carotenoids in chicken tissues [85]. Aflatoxicosis and its masked forms have detrimental effects on poultry, including fatigue, loss of appetite, reduced growth, decreased feed efficiency, lowered egg production, and increased mortality. These toxins also lead to body weight loss, suppressed immune system function, liver dysfunction, blood coagulation disorders, and a significant reduction in jejunal mucosa lutein content by 35% and serum lutein content by 70% in young birds. This suggests that AFB1 and AFBO hinder the absorption, transport, and storage of carotenoids. Additionally, acute aflatoxicosis has been reported as a cause of numerous deaths in waterfowl [85,86,87].

The decrease in feed consumption rates due to mycotoxins can be attributed to various morphological phenomena. One example is the development of mouth ulcers, which can affect the soft and smooth area of the mouth. These ulcers can worsen and lead to infections, causing pain for the bird. Furthermore, the size of the mouth opening can be affected, resulting in a narrower opening. This, combined with the large size of the feed grains, makes it difficult for the bird to swallow the feed. As a result, the bird becomes reluctant to eat, negatively impacting its nutritional efficiency. The outcome of mycotoxin exposure can vary depending on several factors. These factors include the specific type of mycotoxin present, the type of transformed metabolite that is formed, the presence of conjugates if multiple mycotoxins are involved, the rate of infection or the concentration of mycotoxins in the feed, and the age of the bird at the time of infection. It is also important to consider whether there are other substances in the feed that could interact with the mycotoxins. For instance, masked mycotoxins, which are produced when the original toxin is modified, can interact with certain organic acids or other compounds in the feed formula. This interaction can have a negative impact on important production traits, such as growth and immune performance, ultimately leading to significant changes in the bird’s physiological and morphological state and overall health. Hence, it is imperative to carry out further investigations and examinations in order to delve into the impact of mycotoxins and their concealed variations on detrimental alterations in the morphology and anatomy of the digestive system. Consequently, this has a subsequent influence on the nutritional efficacy of poultry.

### 3.2. Deoxynivalenol (DON)

*Fusarium species* generate a diverse mixture of mycotoxins called trichothecenes. The predominant trichothecenes is deoxynivalenol (DON), which is also referred to as vomitoxin. *Fusarium graminearum* and *Fusarium culmorum* are the primary producers of this mycotoxin [88]. The ingestion of contaminated food by animals can lead to immune dysregulation, chronic autoimmune diseases, and abnormal intercellular signaling due to the inhibitory effects of DON on protein synthesis [89] and the binding to sulfhydryl groups at various levels within the subcellular, cellular, and organic systems [28,90]. The high concentration of DON in the external parts is worrisome due to the fact that these parts are commonly utilized for animal feed [91]. The inclusion of DON (5 mg/kg of feed) in broiler chicken diets was found to lead to a decrease in feed consumption [92]. The presence of elevated levels of DON in poultry has been found to have detrimental effects on various aspects such as growth rate and feed conversion efficiency (Table 2). A decrease in feed intake (FI) among animals has been observed as a negative consequence of DON contamination in their diets. The extent of this reduction in FI is contingent upon the levels of contamination present [93]. Broiler chicks fed diets with more than 10% DON- contamination saw declines in broiler chicken body weight (BW), body weight gain (BWG), and feed efficiency (FE) [21]. The inclusion of DON (5 mg/kg of feed) in broiler chicken diets was found to lead to a decrease in feed consumption [92]. A decrease in feed intake (FI) among animals has been observed as a negative consequence of DON contamination in their diets. A reduction in body weight was observed in broiler chickens that were fed a diet artificially contaminated with 5000 μg/kg of DON [94]. However, Keçi et al. demonstrated that dietary contamination with 2500 μg/kg of DON is sufficient to negatively affect bone mineralization in chickens [95]. Their study showed that the impaired growth rate has been linked to the presence of DON, causing toxicity. The inclusion of DON-contaminated diets, even at relatively low to moderate levels (1680 to 12,209 µg/kg feed), led to a decline in both body weight (BW) and the rate of BW gain in broiler chickens [94] (Table 2). The toxic effects of DON in poultry are influenced by various factors, including the dosage and duration of exposure to DON. Additionally, interactions with other dietary components that impact intestinal digestion and overall health appear to contribute to these effects [96]. Immunocompromised broiler chickens exhibited a decline in antibody levels against IBV, which was directly proportional to the dosage of DON present in their feed. This decrease in antibody titers was observed in chickens consuming feed with DON levels ranging from 1680 to 12,209 µg/kg [97]. DON, which is mycotoxin, exhibit limited absorption and tend to persist in the gastrointestinal tract. This persistence leads to the impairment of rapidly dividing intestinal cells and creates an environment conducive to the growth of pathogens within the digestive system [35]. Moreover, prolonged exposure to DON is primarily associated with the disturbance of redox equilibrium, signaling pathways, and the dysregulation of antioxidant defense mechanisms [41]. Hence, additional research is required to comprehensively comprehend the molecular mechanisms underlying the disruptive impacts of mycotoxins on the antioxidant defense network. Furthermore, it is essential to explore viable strategies that can effectively mitigate the nutritional stresses induced by mycotoxins in poultry production. Therefore, further investigation is necessary to gain insights into these aspects and develop efficient solutions. Tissue damage has been associated with alterations in creatine kinase activity, and a decrease in creatine kinase activity was noted in broiler chickens that were fed DON at a concentration of 5000 µg/kg feed [98]. The well-established knowledge regarding the negative effects of DON contamination on poultry’s gastrointestinal health is widely acknowledged [99,100]. According to earlier research, mycotoxins have the potential to affect the weights of organs (Table 2). The tissues that are most vulnerable to trichothecenes mycotoxicosis, particularly DON, are those that have high rates of protein turnover. These tissues include the immune system (bone marrow, lymph nodes, spleen, and thymus), the liver, the intestinal mucosa, and the small intestine [98]. Contrasting findings indicate that broiler chickens fed DON at low to moderate levels (2500 to 10,000 µg/kg feed) exhibited decreased liver weights, as reported in various studies [94]. The harmful effects of Deoxynivalenol toxicity extend to immune organs, including the spleen and thymus, leading to damage, as well as causing changes in the structure of the intestines [101]. The findings of the study [98,102] revealed that broiler chickens, which were fed DON at concentrations ranging from 5000 µg/kg feed to 15,000 µg/kg feed, exhibited an augmentation in the weights of their thymus and spleen.

Broiler chickens exhibit a heightened sensitivity towards DON, which can be attributed to the possibility of DON undergoing conversion into different forms, such as masked DON (Figure 1), during processes involving high heat. It is believed that these transformed versions of DON pose a greater toxicity risk to broiler chicks [119]. Enzymatic conjugation with glucose allows for the transformation of DON into different configurations, as it forms Deoxynivalenol-3-β-D-glucoside (D3G) in a variety of ways [120] or During the process of DON deacetylation, it is possible for the biosynthetic precursors of DON, which are 3 Acetyl-deoxynivalenol (3-ADON) and 15 Acetyl-deoxynivalenol (15-ADON), to be expelled from the system [24,120,121]. Historically, mycotoxins have been assessed and regulated primarily at the feed level. Nevertheless, feed analysis is associated with significant drawbacks. Modified or conjugated forms, formerly known as masked mycotoxins, have the ability to revert back to their free forms, thereby contributing to the detrimental effects associated with mycotoxin exposure. This phenomenon has been observed in broiler chickens for 3- and 15-acetyldeoxynivalenol (3AcDON and 15AcDON, respectively), as well as DON-3-glucoside (DON3G) [122]. Hence, both ADONs and DON3G can be considered equally toxic as DON itself. Detecting these modified forms in feed can be challenging and may not always be achievable using conventional methods that primarily focus on analyzing the non-modified mycotoxin.

The primary indications of DON and its masked forms include vomiting (which led to its nickname, “vomitoxin”), refusal to consume feed, skin damage, and hemorrhaging [2], poultry exposed to feed contaminated with less than 5 mg/kg^−1^ of DON experience a reduced immune response and an increased susceptibility to infectious diseases. Similarly, Santos et al. it was determined that broiler production is impacted when feed is contaminated with masked forms of DON, even at levels categorized as Low (25.6–39.4 μg/kg of 3+15 acteyl-DON), Moderate (42.3–49.1 μg/kg), and High (58.4–71.1 μg/kg), as well as DON-3-Glucoside (Low: 356–362 μg/kg; Moderate: 405–637 μg/kg; and High: 625–787 μg/kg), all of which are below the EU maximum recommendation of 5000 μg/kg. The study reported a notable increase in feed intake among broiler chicks that were fed a contaminated diet. However, this increase was accompanied by a significant impairment in feed conversion ratio (FCR) [123]. Also, Broiler chickens fed a naturally contaminated diet, containing 7500 μg/kg of DON levels combined with one of its masked form (3-Acetyl-DON-1481 μg/kg), exhibited gut leakage [60]. The study of Kolawole et al. revealed that over 50% of diets utilized for feeding broilers contain the DON derivatives 3-Acetyl-DON (3-Ac-DON) and DON-3-glucoside (DON-3-G), with average concentrations of 42.1 μg/kg and 46.5 μg/kg, respectively [90]. 3 + 15Ac-DON and DON-3-G are frequently detected in feedstuffs contaminated with high levels of DON, as 3 + 15 Ac-DON is produced by the same *Fusarium fungi* that produce DON [124]. This masked mycotoxin exhibits increased toxicity, as gizzard erosion is already observed in broilers when orally exposed to 3000 μg/kg [125]. As of now, no specific maximum recommendations have been established for masked forms of DON, 3 + 15 Ac-DON and DON-3-G, and there is still insufficient information regarding their impact on broiler health and performance.

### 3.3. Ochratoxins

*Aspergillus ochraceus* and *Penicillium verrucosum* are the primary sources responsible for the production of Ochratoxin A (OTA), which is the most prevalent and highly toxic variant of ochratoxin. There are many subtypes and masked forms within the ochratoxin family Figure 1. There are three forms of metabolites for ochratoxins, namely ochratoxin A (OTA), ochratoxin B (OTB), and ochratoxin C (OTC). OTA is a toxic mycotoxin that is produced by *A. carbonarius*, *A. niger*, *A. ochraceus* and *P. verrucosum*. Both *Aspergillus* and *Penicillium species* release all forms of ochratoxins. Ochratoxins, particularly Ochratoxin A (OTA), are highly dangerous mycotoxins that pose a significant threat to the productivity and well-being of poultry birds are presented in Table 3. These toxins can be found in various climates, including warmer regions like A. ochraceous and colder regions like P. verrucosum [126]. Unlike aflatoxins, ochratoxins have been found to cause more toxicity in broilers and layers [127]. The toxicological profile of Ochratoxins classifies them as carcinogenic, nephrotoxic, teratogenic, immunotoxic, and mutagenic. The undesirable effects of ochratoxins on poultry feed are twofold: they adversely affect the growth and quality of eggs and meat, and they also lead to economic losses by impacting the bird’s productive performance. Furthermore, these toxins cause histopathological changes in the organs of birds, which further deteriorates their overall health [127].

Studies have indicated that OTA toxicity can have deleterious effects on the gastrointestinal tract of chickens, leading to impaired nutrient absorption and consequently hampering their growth rate (Table 3). Broiler chickens that were fed OTA at levels of 20 or 50 µg/kg BW exhibited a decrease in both body weight (BW) and BW gain [130,137]. A study of Solcan et al. [130] revealed that administering OTA at doses of 20 or 50 µg/kg body weight per day resulted in a decrease in leukocyte counts among young broiler chickens. The mortality rates were highest in broiler chickens that were fed 1000 µg OTA/kg feed and exposed to coccidia, as reported by OTA. Additionally, young broilers that consumed 800 µg OTA/kg feed experienced a mortality rate of approximately 13% [72]. In their study, Li et al. examined the impact of incorporating OTA into the broilers’ diet at a rate of 50 g/kg. They observed that the inclusion of OTA led to an increase in MDA levels in the kidneys, while the total antioxidant capacity (TAC) decrease. Additionally, the levels of SOD, CAT, and GSH were significantly lower compared to the control group. These findings suggest that OTA triggers the generation of reactive oxygen species, leading to oxidative stress in the kidneys of chickens [134]. In his study, Kovesi et al. [133] conducted an assessment on the impact of ochratoxin A (OTA) exposure, specifically at concentrations of 106, 654, and 1126 µg/kg feed, for a duration of 3 weeks. The focus of the evaluation was on various factors including lipid peroxidation, GSH concentration, and GPx activity. Additionally, examined the expression of genes related to oxidative stress response (KEAP1, NRF2) and the glutathione system (GPX3, GPX4, GSS, GSR) in chickens Table 3. The cause of the changes in GSH remains uncertain, as it is unclear whether they are solely a result of an imbalance between GSH synthesis and usage, or if there is also a redistribution of this antioxidant to other tissues as a consequence of OTA intoxication. Throughout the duration of OTA exposure, there was no consistent increase observed in either GSH content or GPx activity. Interestingly, there was a distinct tissue-specific response to OTA, as indicated by the upregulation of the KEAP1 gene in the liver and its downregulation in the kidney. The liver and kidney showed a noticeable increase in the expression of the NRF2 gene when exposed to high levels of OTA. This indicates that the AO system dysregulation is quite complex, as it is further confirmed by the down-regulation of Nrf2 dependent genes, such as GPX3, GPX4, GSS, and GSR, due to OTA exposure. These findings highlight the variations in gene expression and protein synthesis levels of different antioxidants under the stress conditions induced by OTA [138]. At concentrations of 100 µg/kg feed, the presence of OTA resulted in elevated weights of the hearts in broiler chickens, causing toxicity [131].

Despite minor structural differences in functional groups, the ochratoxins exhibit significant variations in toxicity, with OTA being the most potent [139]. An amide bond connects dihydrocoumarin and L-β-phenylalanine in its structure [140]. OTA’s toxicity primarily stems from the presence of a chlorine atom in the dihydro-isocoumarin ring. In contrast, OTB has a hydrogen atom in place of the chlorine atom, resulting in its toxicity being only one-tenth of OTA. Similarly, OTC, Otα, and OTβ do not display notable levels of toxicity [141].The European Commission offers guidelines regarding the maximum allowable levels of OTA in animal feeds. Specifically, for broiler chickens, the concentration of OTA in complete feed should not exceed 0.1 mg/kg, while in individual cereal ingredients used for feed formulation, it should not exceed 0.25 mg/kg [142].

Studies have demonstrated that OTA exhibits its toxic effects by inhibiting mitochondrial function, inducing elevated oxidative stress, and impeding protein synthesis. Additionally, at a molecular level, the detrimental effects of OTA involve the impairment of membrane lipids, DNA mutations, and the nitrosylation of proteins [141]. Numerous research studies have demonstrated that in poultry, OTA has the ability to compromise the integrity of the intestinal barrier by suppressing the expression of TJ proteins including occludin, zonula occludens-1, and claudin-1 [143,144,145]. OTA has the potential to disrupt tight junctions (TJs) by reducing the expression of genes and proteins associated with TJs and elevating the levels of lipopolysaccharides in the bloodstream. This disruption leads to an increase in gut permeability. Consequently, harmful substances, including both commensal and pathogenic bacteria, may translocate from the gut lumen to the internal environment, posing potential harm to animals [146].

OTA is a fungal-derived compound that possesses immunomodulatory properties. According to a study, OTA has been found to stimulate the release of proinflammatory cytokines, including IL-1b and tumor necrosis factor a, in the jejunum of Pekin ducklings. Additionally, OTA decreases the production of the anti-inflammatory factor IL-10 and reduces the content of IgA in the jejunum [143]. Tong et al. revealed that when 1-day-old broiler chickens were fed with 50 mg of OTA per kilogram of body weight, there was an observed upregulation of IL-1b and tumor necrosis factor a mRNA expression. Additionally, the activation of nuclear factor kB through phosphorylation was observed. The broiler chickens in the OTA group exhibited notable signs, including a congested lamina propria in the intestine, infiltration of lymphocytes, presence of necrotic epithelial cells, and varying degrees of shortened, thickened, and edematous intestinal villi [147]. The outcomes of the study indicated that OTA exhibits cytotoxic effects on both the intestinal epithelium and mucosa-associated lymphoid tissue. This cytotoxicity leads to alterations in the integrity of the intestinal barrier and enhances the susceptibility to a range of associated diseases.

### 3.4. Fumonisins

*Fumonisins*, a group of over 25 mycotoxins, are synthesized by fungi belonging to the genus *Fusarium*. Among these mycotoxins, fumonisin B1 (FB1) and fumonisin B2 (FB2) Figure 1, are the most prevalent [148]. Maize seeds exhibit the highest vulnerability to infection, although these mycotoxins can also be detected in sorghum, wheat, barley, and soybeans. Maize seeds are predominantly affected by these mycotoxins, making them the prevailing mycotoxins in this particular crop [149]. FB1 has the potential to induce a range of illnesses in poultry presented in Table 4. Toxic reactions can manifest in different ways, such as decreased weight gain, higher mortality rates, diminished size of the bursa of Fabricius, thymus, and spleen, myocardial degeneration, myocardial hemorrhage, disruptions in the hemostatic mechanism, and hepatocyte necrosis. These adverse effects have been observed in chickens, ducks, and turkey chicks. Consequently, this mycotoxin not only poses a significant risk to human and animal well-being but also compromises food safety and hampers animal production [150]. Animals exposed to fumonisins have shown a correlation with kidney and liver toxicity [151]. The toxicity of FB in animals presents a paradox when it comes to its toxicokinetics during the onset of mycotoxicosis. Previous studies have shown that FB toxicity accumulates over time in avian species such as ducks and turkeys, with prolonged exposure to low doses resulting in an increase in sphinganine (Sa) and sphingosine (So) bases in the liver. These markers are recognized as indicators of FB exposure and toxicity. However, toxicokinetic studies conducted on both avian species and mammals have indicated that FB is rapidly eliminated from the bloodstream, suggesting that its persistence in animals is negligible [152]. This apparent contradiction between the cumulative toxicity of FB and its rapid elimination from plasma may be attributed to the insensitivity of the analytical methods used in these studies [26].

Diets that are tainted with FBs have been found to have negative effects on performance Table 4, leading to issues such as feed refusal and diarrhea.

Experimental studies have shown that even at levels as low as 10,000 µg/kg feed, FBs can have detrimental effects [158]. Furthermore, Li et al. observed that the inclusion of 200,000 µg FB1/kg feed in the diet resulted in a decrease in lymphocyte count in chickens. This immunosuppressive effect was particularly observed in broiler chickens [159]. Likewise, the toxicity of FBs (100,000 or 200,000 µg/kg feed) resulted in increased total plasma protein and albumin levels in broiler chickens [51] additionally, broiler chickens that were fed diets containing FBs at concentrations of 100,000 or 400,000 µg/kg experienced increased cholesterol levels. Furthermore, the toxicity of FBs (200,000 µg/kg feed) with increased serum AST levels in broiler chickens [160].

FUM refers to a class of toxins produced by *Fusarium fungi*. When feed is contaminated, the symptoms associated with FB1 and its masked forms partially hydrolyzed metabolites pHFB1a and pHFB1b, and fully hydrolyzed metabolite (HFB1), include reduced appetite Figure 1. Furthermore, these toxins have been observed to cause morphological changes, such as a decrease in villus height, in broiler chicks [161]. FBs and their masked toxic forms primarily target the liver, kidneys, and intestinal tract in most animal species [162]. Studies have shown that even at low to moderate dietary levels, FBs have detrimental effects on enterocyte viability and proliferation, as well as the production of pro-inflammatory cytokines. These toxins also disrupt the intestinal barrier function, thereby increasing the vulnerability of avian species to significant enteric infectious diseases like coccidiosis and necrotic enteritis [161]. This finding strengthens the hypothesis that FB1 undergoes a conversion into its masked form HFB1 within the intestine and exhibits detrimental effects even at low levels of contamination. Studies have demonstrated the cytotoxicity of HFB and their ability to inhibit sphingolipid synthesis. Among these compounds, FB is the most prevalent and exhibits the highest level of toxicity [163]. In Antonissen et al.’s study, chickens exhibited no pathological signs of toxicity from FBs (fumonisins) and HFB1 (hidden fumonisin B1). There were no differences in body weight between the birds fed a diet contaminated with FBs and the control group. This lack of toxicity can be attributed to the pharmacokinetic nature of HFB1, which is efficiently eliminated from the body. Interestingly, even when broiler chickens were orally exposed to HFB1-contaminated feed, only low levels of the masked metabolite pHFB1 were detected, while no second-phase metabolites or N-acyl metabolites of HFB1 were found [33].

### 3.5. Zearalenone

Previous studies demonstrating that broiler chickens exposed to ZEN experienced a decrease in body weight gain body weight gain are presented in Table 5, [10,12,63,164]. When broiler chickens were fed diets contaminated with ZEN at a concentration of 2000 µg/kg feed, they exhibited a reduction in body weight gain and an increase in feed conversion ratio [137]. In broiler chickens, the administration of subclinical amounts of ZEN, not only increased the severity of coccidiosis but also hindered the recovery process [35]. Furthermore, the toxicity of FBs (200,000 µg/kg feed) or ZEN (2000 µg/kg feed) was associated with increased serum AST levels in broiler chickens [160]. While ZEN has limited reproductive toxicity in poultry, research indicates that it exhibits greater toxicity towards other organs in poultry. This is due to the presence of estrogen receptors (ER) in various tissues and cells, including the uterus, breast, liver, and immune cells, which are regulated by estrogen [165]. The inclusion of ZEN at a concentration of 2 mg/kg can lead to impaired growth performance and the development of achondroplasia in broiler chickens, as well as an increase in liver weight [160]. Adult hens exhibited changes in their serum levels of aspartate aminotransferase (AST) and serum alkaline phosphatase (ALP) following the administration of 0.4 mg/kg of ZEN [102]. Growing layers demonstrated elevated serum levels of low-density lipoprotein (LDL) and cholesterol [166]. The administration of ZEN at a concentration of 2 mg/kg resulted in a decrease in total protein levels, albumin, and antioxidant enzymes in the serum of broiler chickens. Additionally, there was an increase in the levels of AST and ALT (Alanine aminotransferase) [167]. It was discovered that ZEN concentrations exceeding 5 mg/kg led to an increase in the average feed-to-egg ratio among laying hens and triggered significant inflammation [168]. The study findings indicated that the inclusion of 250 μg/kg of ZEN in the feed did not result in any significant effects on feed intake, egg production, or egg quality. However, when the feed contained 750 μg/kg of ZEN, a significant increase in feed conversion ratio (FCR) was observed. Furthermore, the addition of 750 μg/kg of ZEN led to a decrease in albumen height and Haugh unit. Both 250 μg/kg and 750 μg/kg of ZEN supplementation resulted in a reduction in ovarian index, ovarian tissue damage, and dysregulation of reproductive hormones [169]. Broiler chickens can be exposed to a mixture of multiple mycotoxins in their feed and feed ingredients, leading to potential interactions between mycotoxins, such as deoxynivalenol and aflatoxin B1 [170]. According to Chen et al., it was discovered that simultaneous exposure to ZEN leads to damage in the liver and jejunum, along with a decrease in colon weight in chickens [10]. Poultry possess a higher capacity for excreting ZEN and its metabolites due to their faster hepatic and enteric circulation, as well as enhanced excretion capabilities [17]. The reduced sensitivity of poultry to ZEN can be attributed to several factors, including the modulation of intestinal microorganisms, variations in hydroxysteroid dehydrogenase activity, elevated estrogen levels, and decreased affinity of estrogen receptors. These additional components enhance our comprehension of why poultry may display diminished sensitivity towards ZEN.

Zearalenone, alternatively referred to as F-2 mycotoxin, is a metabolite with nonsteroidal estrogenic properties that is synthesized by certain species of *Fusarium* and *Gibberella*, including *Fusarium graminearum* [173,174]. Various metabolites with distinct levels of toxicity can be found in feed, contributing to the presence of ZEN [174,175]. Zearalenone (ZAN), α-zearalenol (αZOL), β-zearalenol (βZOL), α-zearalanol (αZAL) and β-zearalanol (βZAL) are the primary metabolites of this compound Figure 1. High levels of consumption of ZEA have been proven to cause hormonal and estrogenic effects, leading to infertility, particularly in pigs. Poultry demonstrate notable tolerance to ZEN, potentially attributed to the inherent high concentration of estrogen naturally present in their blood [28]. Animals exposed to fumonisins have shown a correlation with kidney and liver toxicity [151]. ZEA possesses a chemical composition comprising a resorcinol moiety connected to a 14-member macrocyclic lactone ring through a trans double bond. Additionally, it contains two hydroxyl groups, two ketones, and one methyl branch. These structural features enable ZEA to be readily absorbed by the gastrointestinal tract and interact with proteins and lipids within the biological system, thereby exerting its toxic effects [174]. Furthermore, experiments conducted in a controlled laboratory environment have demonstrated that ZEA has the ability to enhance the generation of reactive oxygen species (ROS). This, in turn, leads to the occurrence of oxidative stress, which plays a pivotal role in the genotoxic effects of ZEA. These effects include the induction of DNA damage, disruption of DNA repair mechanisms, alteration of the epigenome in targeted cells, and influence on chromatin structure and non-coding RNA [84,176].

ZEN, along with its modified and or/masked forms such as α-zearalenol-Sulfate, α-zearalenol, α-zearalenol glucoside, β-zearalenol, and β-zearalenol-glucoside Figure 1, is recognized as a contributor to hyperestrogenism, leading to reproductive issues, particularly in poultry breeding [2]. Residues of ZEN and its masked forms can be found in the broiler liver [167]. In poultry, scientists have detected the presence of both ZEN and its masked forms, α-ZEL and β-ZEL, in various tissues and excreta, including blood, liver, kidney, muscle, intestine, and excreta [177,178]. Poultry exhibits a higher tolerance to ZEN and its masked forms compared to pigs, which can be attributed to factors such as the lower absorption rate of ZENs, the rapid elimination of metabolites, and the liver’s production of a higher proportion of α-ZOL [17]. At a specific zearalenone level and detection limit, no traces of ZEN or its masked forms were detected in eggs [169]. ZEN and its masked forms demonstrate estrogenic activity and compete for binding to the estradiol receptor. This competition contributes to weight and morphological alterations in the reproductive organs and leads to disorders within the reproductive system of animals [169]. Finally, the toxicity of modified and masked variations of ZEN, including both extractable conjugated and non-extractable bound forms, has not been sufficiently assessed or evaluated. Considering the scarcity of available studies on ZEN, its masked forms, and their impact on poultry health and productive performance, it is crucial to conduct extensive research in the future to fill this knowledge gap.

## 4. Approaches to Mitigate Mycotoxins in Poultry Diets

Mycotoxin contamination typically arises from the proliferation of molds, including *Aspergillus*, *Fusarium*, and *Penicillium*, on various agricultural commodities such as corn used in feed production. These molds generate mycotoxins as byproducts during their growth and development. Contamination can occur either pre-harvest, with molds colonizing crops in the field, or post-harvest, during storage and processing of harvested crops. Factors such as high humidity, inadequate drying, suboptimal storage conditions, and insect infestation contribute to mold growth and subsequent mycotoxin production. Once these mold-infested crops become feed ingredients, mycotoxins can enter the feed chain, potentially affecting the health and performance of birds that consume the contaminated feed. Therefore, it is essential to implement effective mitigation strategies to ensure the safety and well-being of livestock and prevent potential contamination of food products.

Various strategies can be employed to reduce mycotoxin levels in feed and minimize their harmful effects. These include implementing rigorous quality control measures in the production and storage of feed ingredients [43,179], adopting proper post-harvest practices to limit fungal growth and mycotoxin production [180], utilizing physical methods such as sorting and cleaning to remove contaminated grains [181], and employing feed additives such as adsorbents, enzymes, probiotics, prebiotics, antioxidants, and mycotoxin binders. A comprehensive approach that combines preventive measures, monitoring, and appropriate mitigation strategies is essential for managing mycotoxin contamination and safeguarding animal and human health. The effectiveness of these methods typically relies on several factors, such as the initial levels of contamination, the degree of inactivation achieved, the practicality of regular implementation, the safety precautions involved, and the associated costs. These factors play a significant role in determining the success of mycotoxin mitigation strategies, including their masked forms [36].

### Mitigating Masked Mycotoxins in Poultry Feed Can Be Achieved through the Use of Specific Feed Additives: Here Are Some Strategies to Consider

**Adsorbents:** Utilize adsorbent materials such as activated carbon, clay minerals (e.g., bentonite), and yeast cell walls (e.g., *Saccharomyces cerevisiae*) to bind and immobilize mycotoxins, including masked mycotoxins, preventing their absorption in the digestive tract. The ideal adsorbents should demonstrate enhanced affinity for a broad spectrum of mycotoxins, strong adsorption capacity, and minimal binding to essential nutrients [182]. A new mycotoxin detoxifying agent (MMDA) has been created [16], comprising a modified zeolite (clinoptilolite), *Bacillus subtilis*, *Bacillus licheniformis*, *S. cerevisiae* cell wall, and silymarin. This agent operates through adsorption, biotransformation, hepatoprotection, and immunostimulation mechanisms. previous research studies have investigated the utilization of substances to mitigate the adverse impacts of mycotoxin-contaminated feed. These studies have explored the use of toxin adsorbents, such as beta glucans, zeolite, bentonite, hydrated sodium calcium aluminosilicate, to inhibit or lessen mycotoxin absorption and facilitate their excretion [12,183]. In vitro, hydrated sodium calcium aluminosilicate (HSCAS) exhibits a strong ability to adsorb AFB1 and effectively safeguards chickens from the detrimental impacts of AFs in vivo [184]. The elimination rates of DON using EDRs and adsorbents at a concentration of 5 mg/kg ranged from 56% to 79% and 1% to 36%, respectively. At a concentration of 0.5 mg/kg, the removal rates of ZEN by EDRs and adsorbents were 38% to 69% and 7% to 9%, respectively [185].

**Enzymes:** Incorporate enzymes (e.g., β-glucanase, xylanase, and cellulase) into the feed formulation to break down complex mycotoxin structures and convert masked mycotoxins into their free, more detectable forms, thereby reducing their toxic effects. Recent advancements in the field of biological detoxification have revealed the significant advantages of utilizing biological enzymes for the degradation of mycotoxins. Through continuous research, it has become evident that the application of these enzymes holds great potential in effectively breaking down harmful mycotoxins. According to Li et al. [186], the ability to directly target the harmful composition of mycotoxins in contaminated diets and convert them into less toxic or non-toxic substances is crucial for ensuring the safety of feed foods. Ongoing research on AFB1-degrading enzymes has indicated that these enzymes not only reduce the toxicity of animal mycotoxins but also enhance animal production performance, boost antioxidant enzyme activity, and protect the body against mycotoxin-induced harm when used as feed additives. The protective effects of laccase against AFB1-induced oxidative stress and inflammation have been discovered, as it effectively mitigates liver cell apoptosis and minimizes pathological harm to liver and kidney tissues. Additionally, the application of biological enzymes has shown promise in reducing the detrimental impact of AFB1 on the body. Li et al. found that the combination of thioredoxin (Trx) and MSMEG_5998, an F420H2-dependent reductase (FDR) produced by Mycobacterium smegmatis, can decrease the cytotoxic effects of AFB1 on HepG2 cells by enhancing DNA damage and p53-mediated apoptosis. Fumonisin esterase enzymes possess the ability to degrade FBs, resulting in the formation of less toxic metabolites such as hydrolyzed fumonisin B1 (HFB1) and partially hydrolyzed fumonisin B1 (pHFB1). The inclusion of esterase enzymes in poultry diets has been studied, demonstrating their safety and efficacy in breaking down FBs at concentrations as high as 20,000 µg/kg without any detrimental effects on chickens and turkeys [187]. Furthermore, the Trx connection enhances enzyme activity, offering superior protection compared to the native MSMEG_5998. It has been observed that the enzyme activity of glutathione S-transferase in chickens is responsive to various concentrations of AFB1. The predominant mechanism for neutralizing aflatoxin B1-8, 9-epoxide (AFBO) involves the nucleophilic capture of AFBO by the enzyme glutathione S-transferase [188]. Enzyme degradation reagents proved to be more successful in reducing DON and ZEN contamination in simulated gastrointestinal tracts of pigs and poultry, surpassing the effectiveness of the adsorbent method. This finding indicates that degrading enzymes offer specific benefits in safeguarding the animal gut against DON damage [185]. Nevertheless, further in vivo investigations are necessary to fully understand the role of degrading enzymes in animal protection.

**Probiotics and Prebiotics:** Include probiotics (beneficial microorganisms) and prebiotics (nutrients that promote the growth of beneficial microorganisms) in the feed to enhance gut health and microbial balance. This can aid in reducing the bioavailability and adverse effects of masked mycotoxins. To mitigate the negative effects of mycotoxins, mycotoxin modifiers such as fungi, and bacteria utilized to facilitate the conversion of mycotoxins into less harmful metabolites. Incorporating *Lactobacillus* spp. into the diets of broiler chickens has been observed to alleviate the harmful consequences of AFB1, or masked AFB1, and ZEN, as well as DON [10,114]. The utilization of *B. subtilis* fermentation extract demonstrated a substantial amelioration in immune toxicity and nephrotoxicity among broilers, according to the findings. The ability to provide protection can be credited to the CP and other proteases found in the fermentation extract of *B. subtilis*. These enzymes have the capability to break down OTA, transforming it into a non-toxic form known as OTα [189]. According to the report, certain types of bacteria, including Devosia sp. ANSB714 and B. subtilis ASAG 216, have the ability to not only enhance the negative impacts of DON on pig production, but also effectively decrease the presence of DON in the serum, liver, and kidney [190,191].

**Antioxidants:** Introduce antioxidants (e.g., vitamins C and E, selenium, and natural plant extracts) to counteract the oxidative stress induced by mycotoxins. This can help mitigate the negative effects of masked mycotoxins on animal health and performance. According to the study conducted by Ruan et al., [192] has been discovered that TOXO-XL has the ability to mitigate the adverse effects caused by combined mycotoxins on liver and oviduct performance and health in broiler breeder hens. This is achieved by effectively regulating processes such as redox balance, immunity, and apoptosis. The addition of natural antioxidants to the diet has been found to effectively delay or inhibit feed oxidation, providing protection to cellular membranes, proteins, lipids, and nucleic acids against the toxic effects of mycotoxins [193]. Numerous studies have demonstrated the protective effects of minerals such as zinc and selenium against mycotoxins. A significant observation is that organic selenium and modified glucomannans demonstrated a protective effect against T-2 toxicity-induced antioxidant depletion in avian liver [194]. Research conducted by Xiao et al. [195] has demonstrated that selenium offers a protective effect against ZEN-induced cytotoxicity. By inhibiting oxidative stress and DNA damage and regulating the expression of zinc-associated genes, zinc effectively diminished the cytotoxic effects of OTA [196]. In general, plant extracts have the ability to accelerate the removal of ZEN from the body or decrease its affinity for target organs by enhancing the animal’s metabolic function or reducing its stress levels [17]. The study revealed that silymarin has the potential to mitigate ZEN-induced hepatotoxicity and reproductive toxicity in rats [197].

**Mycotoxin binders:** Incorporate specific mycotoxin binders, such as modified clays or yeast cell walls, into the feed formulation. These binders have the ability to adsorb various mycotoxins, including masked mycotoxins, reducing their absorption and toxicity. The use of bentonite clays it has been shown to protect chickens from the harmful effects of AFs, FBs, AFs, and OTA. Additionally, a detoxifying agent containing a combination of binding clay and modifying enzymes found to be partially alleviate the combined effects of OTA and T-2 toxin in chickens [131]. Yeast cell wall extracts have shown promise in reducing the harmful effects of OTA and providing partial protection against the toxic impacts of AFs, FBs, DON, and ZEN [198]. Zhang’s study revealed that incorporating yeast polysaccharides into a diet contaminated with mycotoxins (AFB1, DON, and ZEN) resulted in improved broiler performance, including increased body weight (BW), average daily feed intake (ADFI), and average daily gain (ADG) (*p* < 0.01). Additionally, it led to a reduction in the feed conversion ratio (F:G) of broilers (*p* < 0.01). Meanwhile, significantly increased the activities of T-AOC and SOD and decreased levels of MDA [41]. Nevertheless, the inclusion of yeast polysaccharides in the diets had a significant positive impact on the growth performance of broilers fed with naturally contaminated mixed mycotoxins. Previous studies indicating that yeast polysaccharide-based adsorbents can mitigate the adverse effects of mycotoxin-contaminated feed on broiler growth performance by absorbing mycotoxins and protecting intestinal health [32,43,199]. The study examined the effects of yeast cell wall on broilers fed a naturally contaminated diet containing AF, DON, ZEN, and fumonisin. The results showed that the yeast cell wall supplementation increased T-SOD activity at 21 days and reduced MDA concentrations in serum at 42 days [200]. In their study, Elliott et al. [201] demonstrated that clay minerals, in particular, could induce various adverse health effects in farm animals, including interactions with micronutrients and veterinary substances. Additionally, other studies [202,203] have documented the limitations of the majority of commercial binders or detoxifiers, noting their selective affinity for a single mycotoxin. Hence, clay minerals cannot be regarded as a reliable substance for effectively reducing or eliminating the toxicity associated with mycotoxins of diverse types and concealed forms.

It is important to note that the effectiveness of feed additives in mitigating masked mycotoxins may vary depending on the specific mycotoxin and animal species involved. Hence, it is crucial to precisely identify the varieties of masked mycotoxins, pinpoint the stage at which transformation into the masked form occurs, and assess the level of their toxicity in poultry. This will enable the selection of the most suitable nutritional additives or the development of alternative materials aimed at minimizing or mitigating the toxicity of these masked forms, while preserving the birds’ metabolic pathways and overall health.

## 5. Conclusions

The symptoms observed in experimental studies resulting from mycotoxin exposure are generally can caused by concentrations commonly present in poultry production farm feed. It is worth noting that these symptoms may also be linked to masked mycotoxins, which can undergo transformation into more toxic forms compared to the original toxins.In conclusion, the potential impact of mycotoxins and their masked forms on poultry health and productivity is a serious concern that demands proactive attention. The ability of these hidden toxins to evade conventional detection methods and exert harmful effects on poultry, including reduced growth performance and feed efficiency, immune suppression, and organ damage, underscores the urgency of comprehensive management strategies. The limited understanding of these masked toxins and their potential impact on poultry health underscores the critical need for in-depth research, advanced analytical techniques, and innovative strategies for mitigating their adverse effects. To safeguard poultry health and productivity, it is imperative for the implementation of effective mitigation measures such as improved feed quality control, mycotoxin-binding agents, and toxin-deactivation technologies. Collaborative efforts among poultry producers, researchers, and regulatory authorities are essential to address these difficulties and advance the development of robust detection methods and detoxification technologies tailored to the specific needs of the poultry industry. By overcoming these challenges, the poultry sector can better safeguard animal health, ensure product safety, and maintain high standards of food security for consumers.

## Figures and Tables

**Table 1:** Exploring the Impact of Aflatoxins (AFB1) on the Health and Productivity of Chickens: A Review of Previous Scientific Findings.

Aflatoxin	Treatment	Experimental Period/Days	Impacts/Chicken Type	Publication Date	References
Feed Efficiency and Growth Performance	5 mg/kg	22-d	Decrease in BWG/Broiler	2001	[45]
	2.5 mg/kg	27-d	Decrease in BWG/Broiler	2005	[46]
	2.5 mg/kg	28-d	Decrease in EQT/Layer	2005	[47]
	0.05, 0.1 mg/kg	42-d	Decrease in FI and BW/Broiler	2006	[48]
	0.2 mg/kg	33-d	Decrease in FI and BWG/Broiler	2006	[49]
	149 µg/kg	14-d	Decrease in FI and BW/Broiler	2019	[50]
	100 mg/kg	28-d	Increase in FCR and Decrease in FI and BWG/Broiler	2012	[51]
	80 µ/kg	7-d	Increase in FCR and decrease in FI and BWG/Broiler	2012	[52]
	250–500 ppb/kg	35-d	Decrease in BW/Broiler	2013	[53]
	2 mg/kg	21-d	Decrease in BW and BWG/Broiler	2015	[54]
	0.5–2 ppm	42-d	Increase in FCR and decrease in DFI and DWG/Broiler	2017	[55]
	500 ppb/kg	35-d	Increase in FCR and decrease in BW/Broiler	2017	[56]
	0.5 mg/kg	56-d	Increase in FCR and MR and decrease in BW and BWG/Broiler	2019	[44]
	0.6 mg/kg	42-d	Increase in FCR and decrease in FI and BW/Broiler	2022	[57]
Antioxidants status	0.05, 0.1, 0.5, 1 mg/kg	45-d	Increase in MDA and decrease in SOD, CAT, G6PD and GSH-Px/Broiler	2005	[58]
	3.4 or 8.2 mg/kg	41-d	Increase in MDA in liver, kidney, serum and decrease in GPx activity in liver tissue/Broiler	2008	[59]
	7.54 mg/kg	21-d	Increase in HIF-1α and HMOX in jejunum and xanthine oxidoreductase in liver/Broiler	2013	[60]
	1 ppm/kg	42-d	Increase in MDA and SOD and decrease in and TAC and CAT/Broiler	2014	[61]
	0.15, 0.3 and 0.6 mg/kg	21-d	Increase in MDA and GSH and decrease in spleen levels of GSH-Px, GR and CAT/broiler	2016	[62]
	1 mg/kg	28-d	Increase in MDA, decrease in liver and serum CAT, GPx, T-SOD, GR and GSTs/Broiler	2017	[63]
	20 mg/kg	42-d	Increase in MDA/Broiler	2022	[64]
Blood parameters	0.8 mg/kg	35-d	Decrease in plasma ALT/Broiler	2004	[65]
	2.5 mg/kg	27-d	Decrease serum TP, ALB, and GLB/Broiler	2005	[46]
	0.05, 0.1 mg/kg	42-d	Decrease in g-GGT, AST, and ALT/Broiler	2006	[48]
	80 µ/kg	7-d	Increase in plasma T.P and decrease in ALP, AST and ALT/Broiler	2012	[52]
Blood parameters	250–500 ppb	35-d	Increase in ALB, direct bilirubin, Ca, and P; decrease in UA, Glu, total bilirubin, ALT, AST, and γ-GT/Broiler	2014	[66]
	2 mg/kg	21-d	Decrease in serum T.P, ALB, Ca, and Glu/Broiler	2015	[54]
	500 ppm/kg	28-d	Decrease in ALB/Broiler	2015	[67]
	500 ppb/kg	35-d	Increase in ALT/Broiler	2017	[56]
	250–500 ppm	21-d	Decrease in Glu, Ca, HDL, Cr and increase in AST, ALT/Broiler	2018	[68]
	20 mg/kg	21-d	Increase in Ca and HDL; decrease in ALT, AST/Broiler	2022	[64]
	0.6 mg/kg	42-d	Increase in ALT and AST/Broiler	2022	[57]
Internal organs	2, 2.5, 5 mg/kg	21-d	Increase weight of liver and changed hepatic histopathology/Broiler	2001, 2005, 2015	[45,46,54]
	0.8 mg/kg	35-d	liver had necrosis along with multifocal portal infiltration/Broiler	2004	[65]
	2.5 mg/kg	28-d	Liver AFB1 residues/Layer	2005	[47]
	0.05, 0.1 mg/kg	42-d	Residues of AFB1 and AFM1 in livers and muscles/Broiler	2006	[48]
	0.2 mg/kg	33-d	Changed hepatic histopathology/Broiler	2006	[49]
	80–285 µg/kg	35-d	Decrease thymus and bursa weights/Broiler	2011	[69]
	80 µ/kg	7-d	Paleness, bleeding, and fragility in the liver Kidney swelling and bursal atrophy/Broiler	2012	[52]
	250–500 ppb/kg	35-d	reduced ileum, duodenum, and jejunum intestinal length/Broiler	2013	[53]
	250–500 ppb/kg	35-d	reduced ileum, duodenum, and jejunum intestinal length/Broiler	2013	[53]
	0.5–2 ppm	42-d	Increase heart weight/Broiler	2017	[55]
	0.5 mg/kg	56-d	Increase weights of liver, spleen and kidney/Broiler	2019	[44]

BW = body weight; BWG = body weight gain; DWG = daily weight gain; GR = growth rate; FI = feed intake; DFI = daily feed intake; FCR = feed conversion ratio; EQT = egg quality traits; MDA = malondialdehyde; SOD = superoxide dismutase; CAT = catalase; G6PD = glucose-6-phosphate dehydrogenase; GSH-Px = glutathione peroxidase; TAC = total antioxidants; GR = glutathione reductase; HIF-1α = hypoxia inducible factor 1 alpha; HMOX = heme oxygenase; T.P = total protein, ALB = albumin, GLB = globulin, AST = aspartate aminotransferase, ALT = alanine aminotransferase; GGT = Gamma-glutamyl transferase; ALP = alkaline phosphatase; Ca = calcium; P = phosphate; UA = uric acid; Glu = glucose; γ-GT = Gamma-glutamyl transferase; Cr = creatinine; HDL = high density lipoprotein.

**Table 2:** Exploring the Impact of Deoxynivalenol (DON) on the Health and Productivity of Chickens: A Review of Previous Scientific Findings.

Deoxynivalenol	Treatment	Experimental Period	Impacts/Chicken Type	Publication Date	References
Feed Efficiency and Growth Performance	4.7 or 8.2 mg/kg	56-d	Increase FI and BW/Broiler	2002	[103]
	Up 14 mg/kg	35-d	Increase in FCR and decreased in FI, BW/Broiler	2003	[104]
	5.9 or 9.5 mg/kg	56-d	Decrease in FI and BWG/Broiler	2004	[105]
	5 mg/kg	21-d	Decrease in FI/Broiler	2006	[106]
	1.5 mg/kg	53-d	Improved in FCR and increase in BW/Broiler	2007	[107]
	Up to 18 mg/kg	21-d	Decrease in FI and BWG/Broilers	2011	[108]
	1 and 5 mg/kg	35-d	Decrease in FI, BW and BWG/Broiler	2011	[109]
	10 mg/kg	35-d	Increase in FCR and decrease in FI, BW, BWG/Broiler	2012	[110]
	1.68 or 12.20 mg/kg	35-d	Decrease in FI and BWG/Broiler	2012	[97]
	2.5, 5 or 10 mg/kg	35-d	Decrease in FI, BW and BWG/Broiler	2017	[94]
	7.9 mg/kg	34-d	Increase in FCR and Decrease in FI and BW and BWG/Broiler	2019	[111]
	5 or 15 mg/kg	42-d	Increase in FCR and decrease in BW/Broiler	2020	[98]
Antioxidant stat-us	100–200 ng/mL	6–48-h	Increase in ROS and MDA, decrease in GSH and SOD in embryo fibroblast DF-1 cells/Broiler	2014	[112]
	10 mg/kg	35	Increase in TBARS in jejunum/Broiler	2014	[99]
	10 mg/kg	20-h	Decrease in SOD in serum and MDA or TBARS in the jejunal mucosa/Broiler	2017	[113]
	19.3 mg/kg	7-d	Increase in TBARS GSH and decrease in ABTS in jejunum/Broiler	2020	[114]
Blood Parameters	3 mg/kg	42-d	Decrease in T.P, mg, T.G, F.G and increase in ALT/Broiler	2006	[115]
	3 mg/kg	42-d	Decrease in T.P, T.G, F.G and increase in ALT/Broiler	2007	[116]
Blood Parameters	2.95 mg/kg	28-d	Decrease in T.P, ALB and increase in ALT, AST and ALP/Broiler	2011	[117]
	10 mg/kg	35-d	Decrease in plasma T.P, T.G and U.A/Broiler	2012	[110]
	10 mg/kg	35-d	Decrease in ALT, Cho, and T.G/Broiler	2016	[100]
	5 or 15	42-d	Decrease in CK and Cho/Broiler	2020	[98]
	15 mg/kg	42-d	Decrease in serum Cho/Broiler	2020	[98]
	5 or 15 mg/kg	42-d	Decrease in HGB and erythrocytes/Broiler	2021	[16]
Internal organs	Up to 14 mg/kg	35-d	Increase relative weight of heart and decrease relative weight of spleen/Broiler	2003	[104]
	5 mg/kg	21-d	Unaffected relative weights heart, gizzard, pancreas, caecum, colon, spleen and decrease in small intestine/Broiler	2006	[118]
	1.68 or 12.20 mg/kg	35-d	Increase relative weights of liver and spleen and decrease relative weights of duodenum and jejunum/Broiler	2012	[97]
	10 mg/kg	35-d	Increase relative weight of gizzard and decrease relative weight of kidney/Broiler	2014	[99]
	2, 5 or 10 mg/kg	112-d	Increase relative weight of spleen/Layer	2017	[102]
	2.5, 5 or 10 mg/kg	35-d	Decrease relative weight of liver/Broiler	2017	[94]
	19.3 mg/kg	8-d	Decrease villi height and intestinal health and increase crypt depth/Broiler	2020	[114]
	5 or 15 mg/kg	42-d	Increase relative weight of gizzard and thymus and decrease relative weight of colon and small intestine/Broiler	2020	[98]

BW = body weight; BWG = body weight gain; FI = feed intake; FCR = feed conversion ratio; ROS = reactive oxygen species; MDA = malondialdehyde; SOD = superoxide dismutase; GSH = glutathione; TBARS = thiobarbituric acid reactive species; T.P = total protein; T.G = triglycerides; F.G = fibrinogen; ALT = alanine aminotransferase; AST = aspartate aminotransferase, U.A = uric acid; Cho = cholesterol; CK = creatine kinase; HGB = hemoglobin.

**Table 3:** Exploring the Impact of Ochratoxin (OTA) on the Health and Productivity of Chickens: A Review of Previous Scientific Findings.

Ochratoxin	Treatment	Experimental Period	Impacts/Chicken Type	Publication Date	References
Feed Efficiency and Growth Performance	0.4 or 0.8 mg/kg	35-d	Decrease in FI and BW/Broiler	2006	[128]
	5 mg/kg	365-d	Decrease in EPR and EW/Layer	2010	[129]
	0.05 mg/kg	28-d	Decrease in BWG/Broiler	2015	[130]
	0.1 mg/kg	42-d	Decrease in BW/Broiler	2016	[131]
	0.5 and 1 mg/kg	35-d	Reduced in FI and GR/Broiler	2021	[16]
Antioxidants status	1 mg/kg	24-h	Increase in MDA, mRNA expression of apoptosis-associated genes, and apoptosis rate; decrease in SOD, and GSH levels/Broiler	2018	[132]
	106,654 and 1126 µg/kg	21-d	Increase in liver and kidney MDA, GSH in blood plasma and liver/Broiler	2019	[133]
	50 µg/kg	21-d	Increase in kidney MDA and decrease in GSH, SOD, CAT (m-RNA expression), SOD (m-RNA expression) and GSH-Px (m-RNA expression)./Broiler	2020	[134]
Blood Parameters	1 mg/kg	1-d	Increase in AST and ALT/Broiler	2018	[132]
	3 mg/kg	10-d	Increase in blood Glu, U.A, ALT and AST/Broiler	2021	[135]
Internal organs	2.5 mg/kg	21-d	An increase in kidney weight/Broiler	1999	[136]
	0.4 or 0.8 mg/kg	35-d	An increase in gizzard mass/Broiler	2006	[128]
	0.05 mg/kg	28-d	Changes in the anatomy of the intestinal mucosa/Broiler	2015	[130]
	0.1 mg/kg	42-d	OTA residues in the liver and increase weight/Broiler	2016	[131]
	3 mg/kg	10-d	Hemorrhages on the epicardium and duodenal mucosa, catarrhal enteritis/Broiler	2021	[135]

BW = body weight; BWG = body weight gain; FI = feed intake; EPR = egg production rate; EW = egg weight; GR = growth rate; MDA = malondialdehyde; GSH = glutathione; SOD = superoxide dismutase; GSH-Px = glutathione peroxidase; AST = aspartate aminotransferase; ALT = alanine aminotransferase; Glu = glucose; U.A = uric acid; OTA = Ochratoxin A.

**Table 4:** Exploring the Impact of Fumonisins (FB1) on the Health and Productivity of Chickens: A Review of Previous Scientific Findings.

Fumonisins	Treatment	Experimental Period	Impacts/Chicken Type	Publication Date	References
Feed Efficiency and Growth Performance	5 mg/kg	22-d	Decrease in BWG/Broiler	2001	[45]
	50,200 mg/kg	33-d	Decrease in BW and BWG/Broiler	2006	[49]
	200 mg/kg	33-d	Decrease in BW and BWG/Broiler	2006	[49]
	100 mg/kg	21-d	Increase in FCR/Broiler	2014	[153]
	100 mg/kg	28-d	Increase in FCR and decrease in FI and BW/Broiler	2016	[130]
Antioxidants status	100 mg/kg	21-d	Decrease in SOD, GST and NPSH	2014	[153]
	2.5, 5, or 10 ppm	11-d	Increase in liver LPO and ROS; decrease in SOD, GPx, and GST activity; decrease in serum LPO levels and ROS levels/Broiler	2020	[154]
	600 mg/kg	15-d	Increase in TPA, ROS, and SOD; decrease in GPx/Broiler	2020	[155]
Blood Parameters	25, 50 or 100 mg/kg	8 -15-d	Increase in plasma ALT and AST, Ur and Cr/Broiler	2017	[156]
	2.5, 5, or 10 ppm	11-d	Decrease in T.P, ALB, Glu, ALT, T.G and U.A/Broiler	2020	[154]
	600 mg/kg	15-d	Increase in Cho and decrease in U.A/Broiler	2020	[155]
	0.05–20 ppm	42-d	Increase in ALT, AST and U.A/Broiler	2021	[157]
Internal organs	50 mg/kg	22-d	Variations in hepatic histopathology/Broiler	2006	[49]
	200 mg/kg	33-d	Increase liver weight and changes in hepatic histopathology/Broiler	2006	[49]
	100 mg/kg	28-d	Reduced villus height and the ratio of villus to crypt Variations in hepatic histopathology/Broiler	2012	[51]
	100 mg/kg	21-d	Increase liver absolut and relative weight/Broiler	2014	[153]

BW = body weight; BWG = body weight gain; FI = feed intake; FCR = feed conversion ratio; SOD = superoxide dismutase; GST = glutathione S-transferase; NPSH = non-protein thiol; OS = reactive oxygen species; ALT = alanine aminotransferase; AST = aspartate aminotransferase; U.A = uric acid.

**Table 5:** Exploring the Impact of Zearalenone (ZEN) on the Health and Productivity of Chickens: A Review of Previous Scientific Findings.

Zearalenone	Treatment	Experimental Period	Impacts/Chicken Type	Publication Date	References
Feed Efficiency and Growth Performance	2 mg/kg	42-d	Increase in FCR and decrease in BWG/Broiler	2019	[160]
	0.05–20 ppm	42-d	Decrease in FI, FCR and WG/Broiler	2021	[157]
Antioxidants status	7.9 mg/kg	14-d	Increase in GSH-Px, and levels of MDA/Broiler	2012	[171]
	0.4 mg/kg	47-d	Increase in GSH-Px and T-SOD/Layers	2016	[166]
Blood Parameters	7.9 mg/kg	14-d	Increase in GGT/Broiler	2012	[171]
	5 to 200 mg/kg	28-d	Disrupted sex hormones b-endorphin, LH, and progesterone, occurrence of renal edema and nephremia and increase levels of urea and creatinine/Layers	2019	[168]
	0.8 mg/kg	35-d	Decrease in T.P and GLB, and increase in ALT/Broiler	2022	[172]
Internal organs	0.4 mg/kg	112-d	Increase relative weight of oviduct and ovary, degeneration and atrophy of the ovarian tissues/Layers	2017	[102]
	2 mg/kg	42-d	ZEN residues in the kidney and liver A rise in liver weight/Broiler	2019	[160]

FCR = feed conversion ratio; BWG = body weight gain; FI = feed intake; GSH-Px = glutathione peroxidase; MDA = malondialdehyde; T-SOD = total superoxide dismutase; GGT = gamma-glutamyl transpeptidase; LH = luteinizing hormone; T.P = total protein; GLB = globulin; ALT = alanine amino transferase; ZEN = Zearalenone.

## Data Availability

Not applicable.
